# Protective effect of syringic acid via restoring cells biomechanics and organelle structure in human lens epithelial cells

**DOI:** 10.1007/s10863-021-09873-9

**Published:** 2021-03-11

**Authors:** Rong Yang, Xue Li, Jie Mei, Wencheng Wan, Xinduo Huang, Qiaohong Yang, Xiaoyong Wei

**Affiliations:** School of Basic Medical Sciences, Guangzhou University of Chinese Medicine, Guangzhou, 510006 China

**Keywords:** Syringic acid, Human lens epithelial cells, Diabetic cataract, Interaction, Biomechanics

## Abstract

We have previously reported that syringic acid (SA) extracted from *D. aurantiacum var. denneanum (kerr)* may be used to prevent diabetic cataract (DC). However, the underlying mechanisms through which SA prevents DC in human lens epithelial cells (HLECs) remained unclear. In the present study, we employed single-molecule optics technologies, including transmission electron microscopy (TEM), atomic force microscopy (AFM), laser scanning confocal microscopy (LSCM) and Raman spectroscopy, to monitor the effect of SA on HLECs biomechanics and organelle structure in real-time. TEM suggested that SA improved the ultrastructure of HLECs with regard to nuclear chromatin condensation and reducing mitochondrial swelling and degeneration, which may aid in the maintenance of HLECs integrity in the presence of glucose. AFM revealed a reduced surface roughness and stiffness following SA treatment, suggesting an improved viscoelasticity of HELCs. Raman spectrometry and LSCM further revealed that these changes were related to a modification of cell liquidity and cytoskeletal structure by SA. Taken together, these results provide insights into the effects of SA on the biomechanics of HLECs and further strengthen the evidence for its potential use as a novel therapeutic strategy for DC prevention.

**Supplementary Information:**

The online version contains supplementary material available at 10.1007/s10863-021-09873-9.

## Introduction

Cataract is a primary cause of visual impairment in diabetic patients and, due to the rising incidence of diabetes, diabetic cataract (DC) constitutes an emerging global health problem (Abdel-Ghaffar et al. 2018; Goutham et al. 2017; Hwang et al. 2019; Peterson et al. 2018). Surgery is currently the main therapeutic option for DC (Boscia et al. 2017), but there is a high risk of complications, including diabetic macular edema, diabetic retinopathy progression, and posterior capsular opacification, associated with cataract surgery in these patients (Yang et al. 2017; Peterson et al. 2018). Therefore, it is critical to investigate novel efficient therapeutic strategies against DC.

Lens epithelial cells (LECs) play an important role in the protection of the lens and the maintenance of lens transparency (Sorkou et al. 2019). Previous research findings suggest that during the development of DC, several pathological processes occur, including, an increased osmotic pressure due to an activation of the polyol pathway, an imbalance of redox state due to disruptions of the oxidative stress pathway, and an accumulation of advanced glycation end-products in the glycosylation pathway. These processes result in the breakdown of cellular structure and disrupt the intracellular homeostasis of LECs (Bhadada et al. 2016; Obrosova et al. 2010), resulting in opacification of the lens (Yang and Zhang 2015). Alterations in cellular mechanics (structure, adhesion, and mobility) may also be associated with increased lens opacity (Maddala et al. 2016). Furthermore, proliferation and migration of LECs have been observed after cataract surgery and have been suggested to cause capsular opacity and other postoperative complications (Wertheimer et al. 2014; Choi et al. 2012).

Syringic acid (4-hydroxy-3,5-dimethoxybenzoic acid, PubChem CID: 10742, SA) is an active ingredient extracted from *D. aurantiacum var. denneanum (kerr),* which is a traditional Chinese medicine herb used for treatment of diabetes (*Shen-ntonz’s Classic of Materia Medica*). We have previously shown that SA can effectively reduce opacification in human lens epithelial cells (HLECs) and may thus be suitable for treatment of DC (Wei et al. 2012; Wu et al. 2016). However, the underlying molecular changes in the structure, behavior and microenvironment of HLECs following SA treatment were unclear so far.

LECs stimulated with high glucose levels exhibit an increased expression of aldose reductase and an activation of the polyol pathway (Kanchan et al. 2016; Petrovič 2014). These processes are highly similar to pathological processes occurring in DC development. Therefore, we used HLECs stimulated with high glucose levels as an in vitro model to explore the effects of SA on structure and behavior of LECs. We applied single-molecule optics technologies, such as transmission electron microscopy (TEM), atomic force microscopy (AFM), and laser scanning confocal microscopy (LSCM) to observe the mechanical structure, adhesion, mobility and other dynamic behaviors in HLECs after treatment with SA. We found that biomechanics of these cells were modified by SA treatment, and these findings are critical for further characterization of SA as a therapeutic for DC prevention.

## Materials and methods

### Reagents

Minimum essential medium (MEM), phosphate buffered solution (PBS) and penicillin-streptomycin and 0.25% trypsin were purchased from Gibco (Grand Island, NY, USA); fetal bovine serum (FBS) was purchased from Biological Industries (Beit Haemek, Israel); glucose was purchased from Aladdin Industrial Corporation (Shang Hai, China); rhodamine was purchased from Enzo Life Sicences (Farmingdale, NY, USA); DAPI (4′,6-diamidino-2-phenylindole) was purchased from Yeasen (Shanghai, China); triton-100 was purchased from Dingguo Changsheng biotech Co. Ltd. (Beijing, China); Syringic acid was extracted at a purity greater than 98% using the method previously described by Zhang et al. (2008). The HLEC line SRA01/04 was a kind gift from the Ophthalmology Center of the Sun Yat-Sen University (China).

### Instruments

The following instruments were used for our experiments: CO_2_ incubators (Scientific, Thermo, German); transmission electron microscopy (TEM, tecnai G2 spilit twin, FEI, Czech Republic).; atomic force microscopy (AFM, Dimension Fast Scan, Bruker, German); Micro-Raman spectrometer (Renishawinvia, Renishaw, England); ZEISS LSM800 LSCM (Carl Zeiss Microcopy GmbH, Göttingen, Germany).

### HLECs culture and treatment groups

SRA01/04 cells were cultured in complete MEM with 10% of FBS and 1% penicillin-streptomycin. After cells reached 80% confluence, cells were digested with trypsin and used for experiments. HLECs were divided into three groups (*n* = 5): a control group (10% FBS in MEM); a model group (50 mM glucose +10% FBS in MEM); SA group (0.5 μM SA + 50 mM glucose +10% FBS in MEM). In the SA group, glucose and SA were added to the medium at the same time.

### Transmission electron microscopy

HLECs were seeded into 60 mm culture plates at a density of 5 × 10^4^ cells/well. After treatment for 24 h, cells were collected for TEM. HLECs were washed with PBS for 5 min and fixed in 2.5% glutaraldehyde in 0.1 M PBS (pH 7.3). After fixation with glutaraldehyde, cells were washed with PBS three times and post-fixed in 1.2% osmium tetroxide in PBS. The cells were then dehydrated with ethanol and acetone, and embedded in EPON. Ultrathin sections (70–90 nm thick) were stained with acetic acid uranium. Finally, ultrathin sections were collected on naked copper grids to be visualized by TEM.

### Atomic force microscopy

Live HLECs were visualized by AFM after treatment for 24 h with the following parameters: scanning speed = 0.2 Hz; cantilever elastic coefficient = 1–3 N/M; approach velocity = 0.5 μm/s; scan size = 30–50 μm^2^ per cell. Cells were observed for 1–2 h in each group, during which cells remained viable (as previously described by Preedy et al. 2014). Surface roughness and stiffness were assessed using cell surface topography images, with 10–15 areas of 5 × 5 μm. The nanoscope analysis software was employed for the analysis of AFM images.

Quantitative parameters of average roughness (Ra) and root-mean-squared roughness (Rq) values were used to investigate the roughness of cells, calculated using formula (1) and (2), respectively. N was the sampling point and Z was the height of Z axis (Lara-Cruz et al. 2016).

1 $$ {\mathrm{R}}_{\mathrm{a}}=\frac{1}{\mathrm{N}}{\sum}_{\mathrm{j}=1}^{\mathrm{N}}\left|{\mathrm{Z}}_{\mathrm{j}}\right| $$

2 $$ {\mathrm{R}}_{\mathrm{q}}=\sqrt{\frac{\sum {\mathrm{Z}}_{\mathrm{i}}^2}{\mathrm{N}}} $$

The stiffness of each cell was investigated using the Young’s modulus (Brochu and Vermette 2008; Pi et al. 2014) and the Hertz’s contact model was used in this experiment. The relationship between the loading force, F in spherical probe, and indentation, δ, could be described by the formula (3) (Alsteens et al. 2008), where R was the radius of the tip and E_r_ was the reduced Young’s modulus. A reduced Young’s modulus E_r_was correlated with the Young’s modulus of simple E_s_, which is described by formula (4) (Alsteens et al. 2008). V_T_ and V_S_ were the Poisson ratio of the tips and samples. The Poisson ratio of cell was assumed to be 0.5 (Nijenhuis et al. 2010).

3 $$ {\mathrm{F}}_{\left(\updelta \right)}=\frac{4}{3}\sqrt{\mathrm{R}}{\mathrm{E}}_{\mathrm{r}}{\updelta}^{3/2} $$

4 $$ \frac{1}{{\mathrm{E}}_{\mathrm{r}}}=\frac{1-{{\mathrm{V}}_{\mathrm{t}}}^2}{{\mathrm{E}}_{\mathrm{T}}}+\frac{1-{{\mathrm{v}}_{\mathrm{S}}}^2}{{\mathrm{E}}_{\mathrm{S}}} $$

### Raman spectroscopy

HLECs were collected by centrifugation at 3000 g for 5 min after treatment, washed three times, re-suspended in PBS, and visualized using the Raman scattering spectra.

A Raman spectral range of 0–4000 cm^−1^and 10–15 spectra per group was used for the Raman spectroscopy (as previously described by Nijenhuis et al. 2010). The excitation wavelength was 633 nm. The spot diameter was 1.5um, and the testing time was 30 s for each sample (three times per sample). An integration time of 90 s and a low laser power (50%) were used for spectral acquisition to avoid cellular damage.

For Raman spectroscopy, the chain in longitudinal order-parameters (S_trans_) and lateral interaction between chain parameters (S_lat_) of membranes were used to further investigate the fluidity of HLECs following SA treatment (previously described by Anthony 1982). S_trans_ and S_lat_ were used to detect changes in the lipid bilayer structure in living cells and a Raman line at 1655 cm^−1^ (CH2-bending mode) showed a sensitive means to monitor the modulation of lipids in biological cells (Bogliolo et al. 2013). S_trans_ and S_lat_ were calculated using the follow equations, respectively (5), (6) (Anthony 1982),

5 $$ {\mathrm{S}}_{\mathrm{lat}}=\frac{{\mathrm{I}}_{\mathrm{CH}2\ \mathrm{control}}-0.7}{1.5} $$

6 $$ {\mathrm{S}}_{\mathrm{trans}}=\frac{\raisebox{1ex}{${\mathrm{I}}_{1130}$}\!\left/ \!\raisebox{-1ex}{${\mathrm{I}}_{1090}$}\right.}{1.77} $$

where I_CH2_ was the ratio of I_2885_/I_2850,_ I_2885_ and I_2850_ showed the symmetrical stretching vibration and anti-symmetric stretching vibration of CH_2_ in the structure of cell membranes; I_1130_, and I_1090_ showed the anti-conformation and twisted-conformation of the framework C-C in phospholipid of membranes; and I_2885_, I_2850_, I_1130_, and I_1090_ were the intensity at the corresponding Raman shift.

### Laser scanning confocal microscopy

HLECs on glass coverslips were washed with PBS and fixed with 95% ethyl alcohol for 2 h after treatment. Samples were permeabilized with 0.3% Triton X-100 for 30 min and washed twice with PBS. Cells were then incubated with rhodamine dye solution for 1 h to stain F-actin in the cytoskeleton, and DAPI was used to counterstain the nucleus. Images were captured using the LSCM.

### Statistical analysis

All data are presented as the mean ± SD of at least three independent experiments. Spss22.0 software was used for the analysis of variance. Comparisons between groups were carried out using the unpaired two-tailed Student`s t-test and one-way analysis of variance (ANOVA). Differences were considered statistically significant when *P* < 0.05.

## Results

### Ultrastructural changes following glucose stimulation are alleviated by SA treatment

**Fig. 1:**
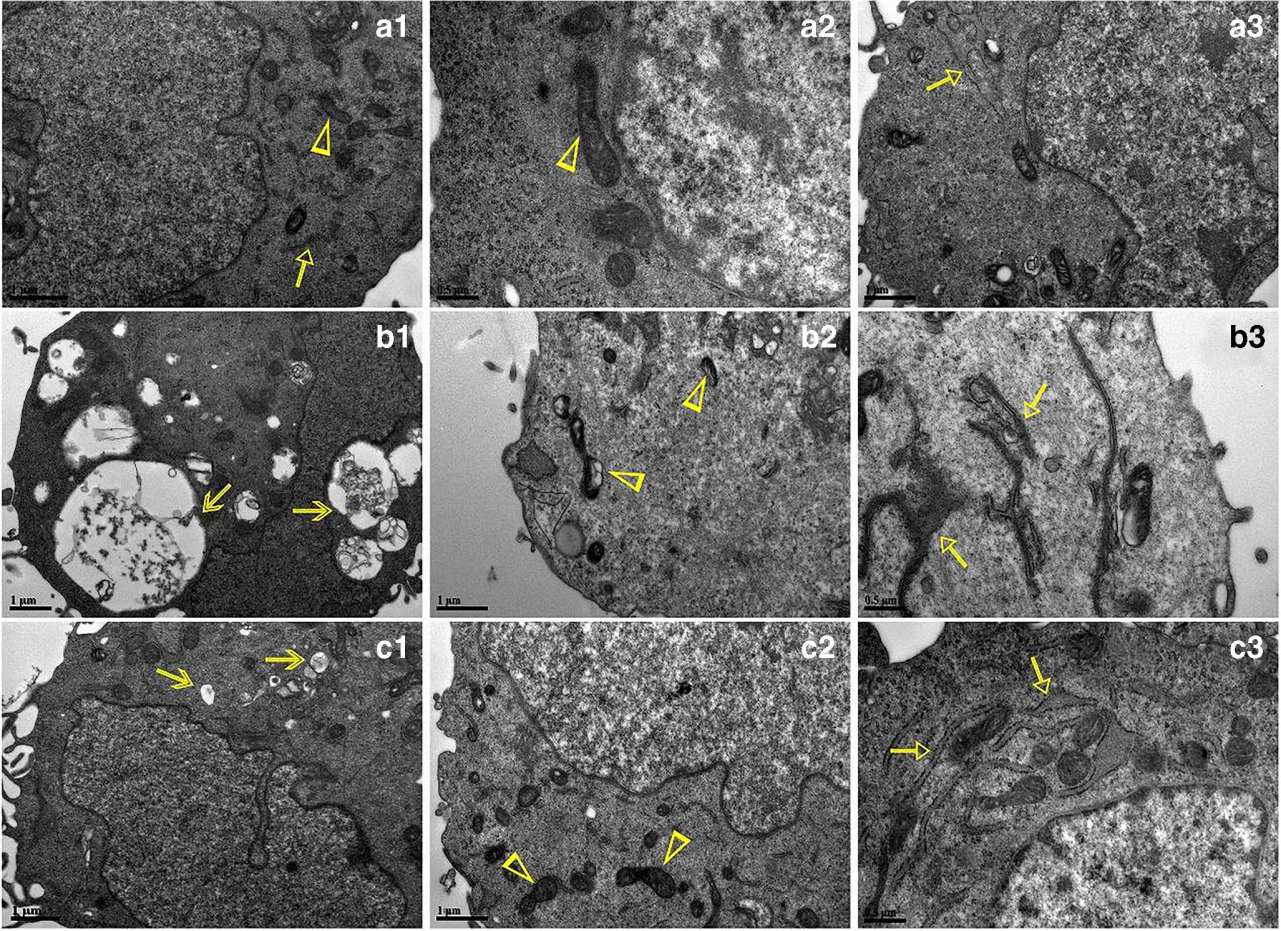
Morphological features of glucose-stimulated HLECs treated with SA under TEM. a1–3 show with normal ultrastructure, characterized by regular mitochondria and endoplasmic reticulum in HLECs; b1–3 show the HLECs induced with 50 mM glucose with obvious vacuoles (b1), abnormal cristae in mitochondria (b2) and endoplasmic reticulum (b3); c1–3 show decreased vacuoles (c1), increased normal cristae in mitochondria (c2) and endoplasmic reticulum (c3). Triangles represent mitochondria, triangle arrows represent endoplasmic reticulum, and V-shaped arrows represent vacuoles

TEM images are shown in Fig. 1. HLECs in the control group showed intact organelles in the cytoplasm, a clearly demarcated membrane, a regular nucleus, and mitochondria with clear and complete cristae (Fig. 1, a1–3). However, in the glucose-induced model group, we observed a highly vacuolated cytoplasm (Fig. 1, b1), deformation of cells, vacuolization and abnormal cristae of mitochondria (Fig. 1, b2), and a swollen endoplasmic reticulum without ribosomes (Fig. 1, b3). Following SA treatment, these changes were reversed, showing an alleviation of cytoplasmic vacuolation (Fig. 1, c1), more definition of mitochondrial cristae (Fig. 1, c2), and rescue of endoplasmic reticulum structure compared to the model group (Fig. 1, c3).

### SA restores membrane integrity of HLECs

**Fig. 2:**
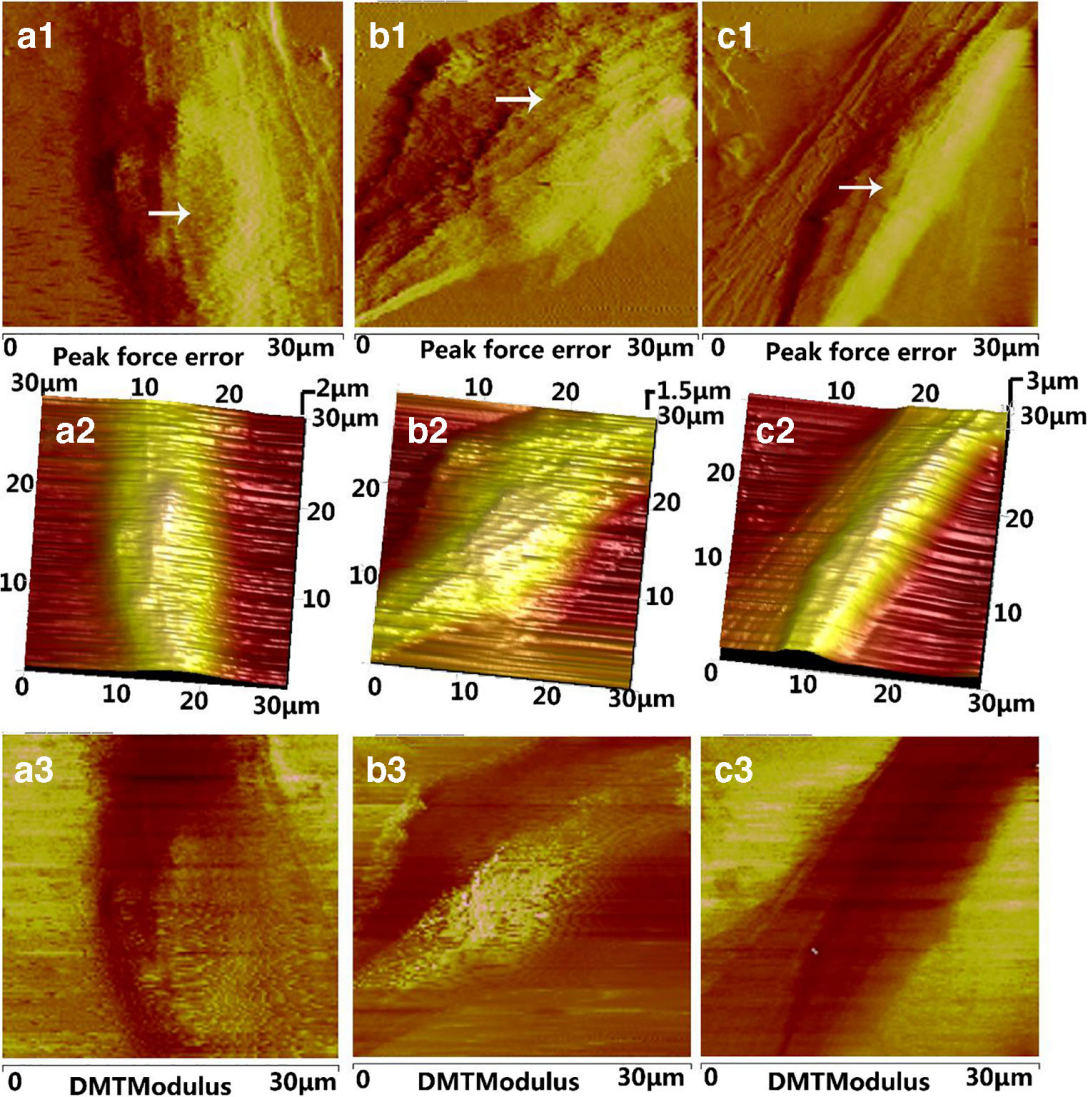
AFM topographic images of normal HLECs (a1–3), 50 mM glucose treated HLECs (b1–3) and SA-treated 50 mM glucose-induced HLECs (c1–3). (a1, b1, c1) peak force error images; (a2, b2, c2) AFM 3-D height images; (a3, b3, c3) DMT Modulus images. White arrows in (a1, b1, c1) indicate the difference in cell membranes between the three groups

**Fig. 3:**
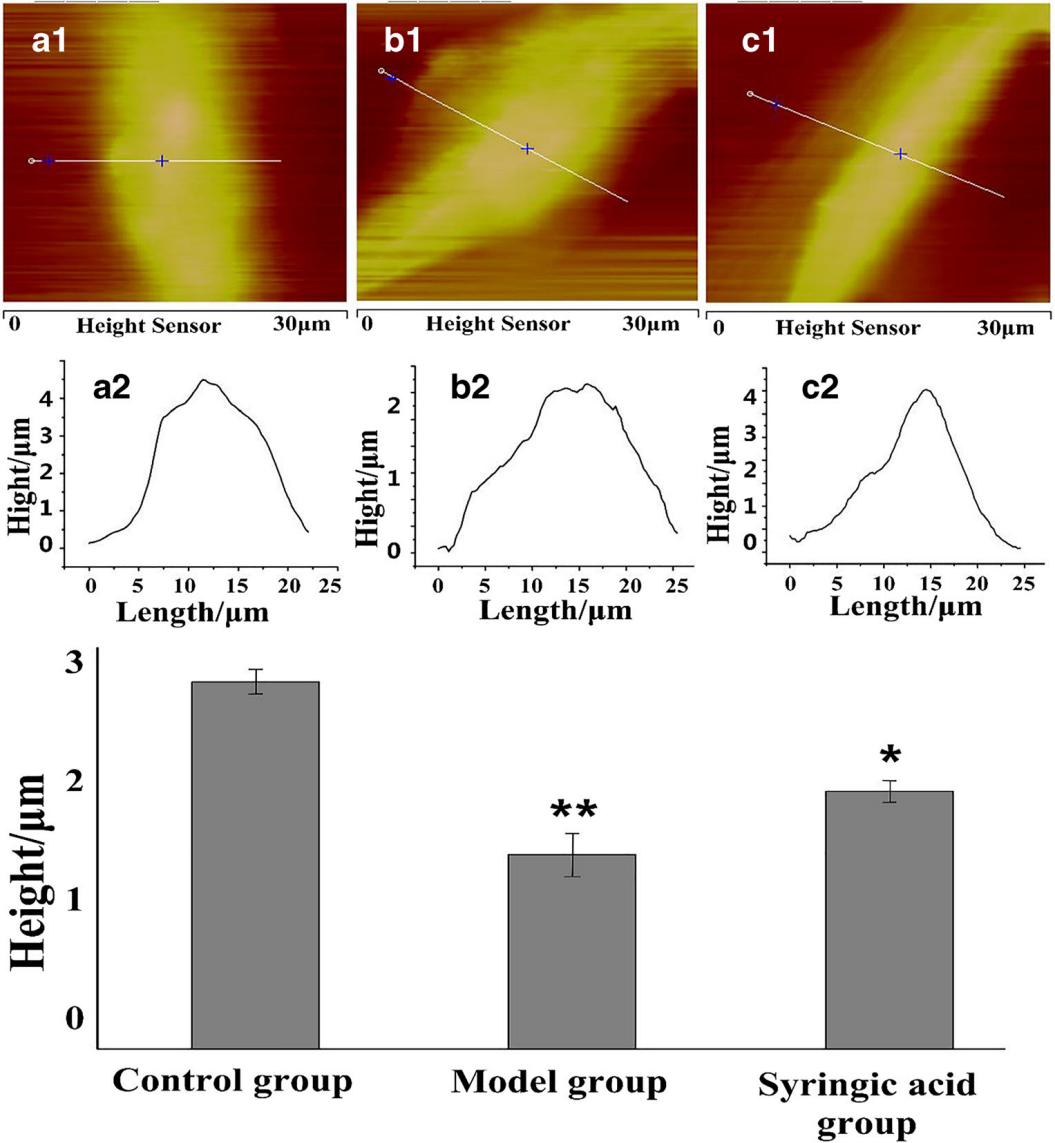
Representative AFM images of normal HLECs (a1–2), 50 mM glucose treated HLECs (b1–2) and SA-treated HLECs stimulated with 50 mM glucose (c1–2). (a1, b1, c1) AFM height images (a2, b2, c2) and height profiles across the section line as marked in a1, b1, c1. The average height of cells for each group is shown in the histogram. ^**^P < 0.01, model group compared to the control group; ^*^P < 0.05, SA group compared to the model group

Morphological changes of HLECs are shown in Fig. 2. In the model group, the surface morphology of HLECs was highly folded compared to the control group (Fig. 2, b1–3), which was decreased by SA treatment (Fig. 2, c1–3). A cross-sectional deformation profile (Fig. 3) revealed a HLEC height of 3.3 ± 0.15, 1.8 ± 0.35, 2.3 ± 0.12 μm in control group, model group, and SA group, respectively. These findings demonstrated that SA could partially restore the height of cells in the presence of high glucose.

### SA reduces roughness and Young’s modulus of HLECs

**Fig. 4:**
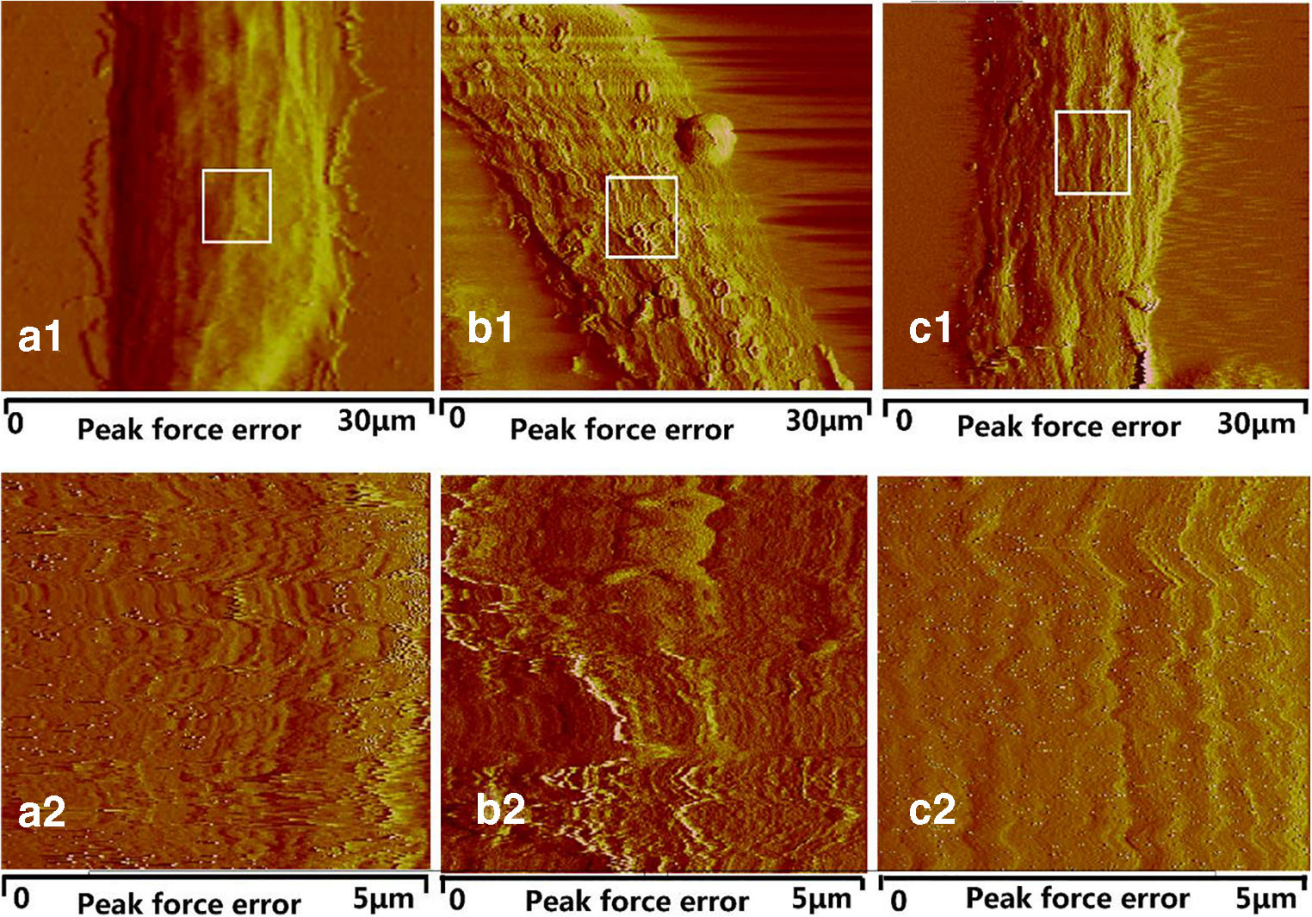
AFM images for peak force error of normal HLECs (a1–2), 50 mM glucose treated HLECs (b1–2) and SA-treated 50 mM glucose-induced HLECs (c1–2). (a1, b1, c1) Scanned area of 30 μm × 30 μm for cells; (a2, b2, c2) Scanned area of 5 μm × 5 μm for cells (enlarged as marked in (a1, b1, c1))

**Fig. 5:**
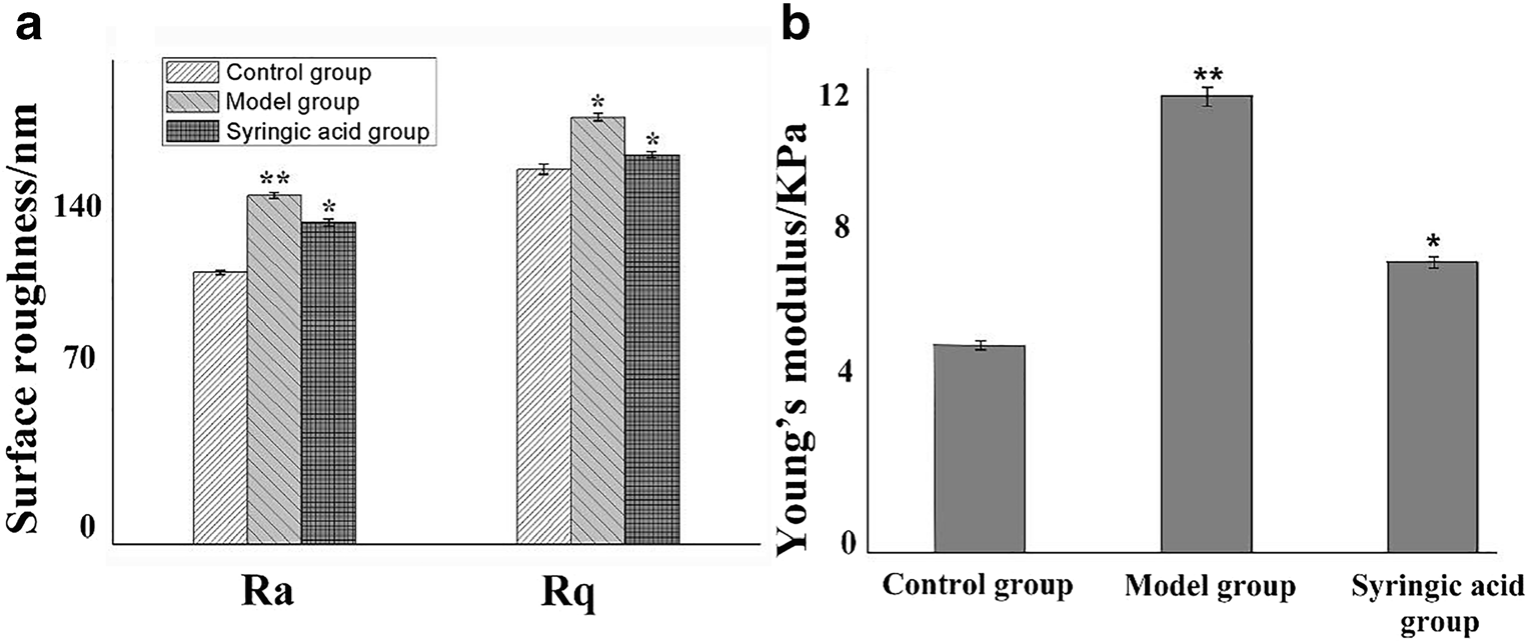
Surface roughness analysis (**a**) and Young’s modulus analysis (**b**) of control group, model group, SA treated group. ^*^P < 0.05, SA group compared with model group; ^**^P < 0.01, model group compared with control group

The Ra and Rq of HLECs were 112.25 ± 2.01 nm and 155 ± 3.07 nm in the control group, 144.2 ± 4.13 nm and 176.6 ± 3.54 nm in the model group, and 132.9 ± 4.56 nm and 161 ± 5.28 nm in the SA group, respectively (Fig. 5 a), as quantified from AFM images (Fig. 4). In the model group, the membrane of HLECs became much rougher compared to the control group (*p* < 0.01, Fig. 4, a1–2, b1–2; Fig. 5a). However, SA treatment significantly reduced HLEC roughness following glucose stimulated in comparison to the model group (*p* < 0.05, Fig. 4, b1–2, c1–2; Fig. 5a). Furthermore, the Young’s modulus was 5.5 ± 0.09 KPa in the control group, 12.1 ± 0.12 KPa in the model group, and 7.8 ± 0.13 KPa in the SA group (Fig. 5b). These findings demonstrated that SA treatment both decreased the cell surface roughness and Young’s modulus of HLECs in the presence of high glucose.

### SA improves membrane liquidity of HLECs following glucose stimulation

**Fig. 6:**
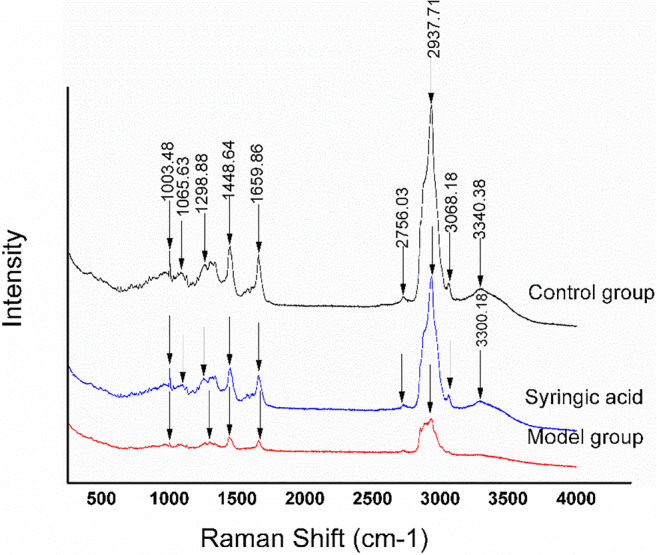
Change of Raman spectra with or without SA treatment. There were three groups (control group, model group, SA group) in this experiment

We used Raman spectroscopy to estimate the lipid composition of the cell membrane of HLECs. The Raman spectrum after 24 h SA treatment in HLECs is shown in Fig. 6. Characteristic peaks appeared at 1003, 1065, 1298, 1448, 1659, 2598, 2937, 3068, 3340 cm^−1^ for interaction time of the control group, and the Raman peak was changed at 2606 cm^−1^ in the model group, and 2756 cm^−1^ in the SA group. Relating to lipids, the CH2-bending mode was related to the peaks at 1448 and 1141 cm^−1^, and the C=C-stretching mode was related to the peaks at 1659 and 1655 cm^−1^.

S_lat_ and S_trans_ were 1.23 ± 0.11 and 0.83 ± 0.09 in the control group, 1.70 ± 0.19 and 0.92 ± 0.18 in the model group, and 1.72 ± 0.16 and 0.84 ± 0.08 in the SA group, respectively (Table. 1). S_trans_ and S_lat_ were significantly increased in the model group compared to the control group (*p* < 0.05), which indicated that glucose increased the order of chains in the lipid bilayer of HLECs and decreased the liquidity of the cytolemma. Compared with the model group, S_trans_ was decreased in the SA group (*p* < 0.05), which suggested that SA could improve cell liquidity, although S_lat_ did not change significantly.

**Table 1:** S_lat_ and S_trans_ after treatment with SA following Raman spectroscopy

Groups	S_lat_	S_trans_
Control group	1.23 ± 0.11	0.83 ± 0.09
Model group	1.70 ± 0.19^**^	0.92 ± 0.18^*^
SA group	1.72 ± 0.16	0.84 ± 0.08^*^

^**^P < 0.01, model group compared with control group; ^*^P < 0.05, SA group compared with model group. S_lat_: I2885/I2850; S_trans_: I1130/I1090

### Cytoskeletal disruptions are alleviated following SA treatment

**Fig. 7:**
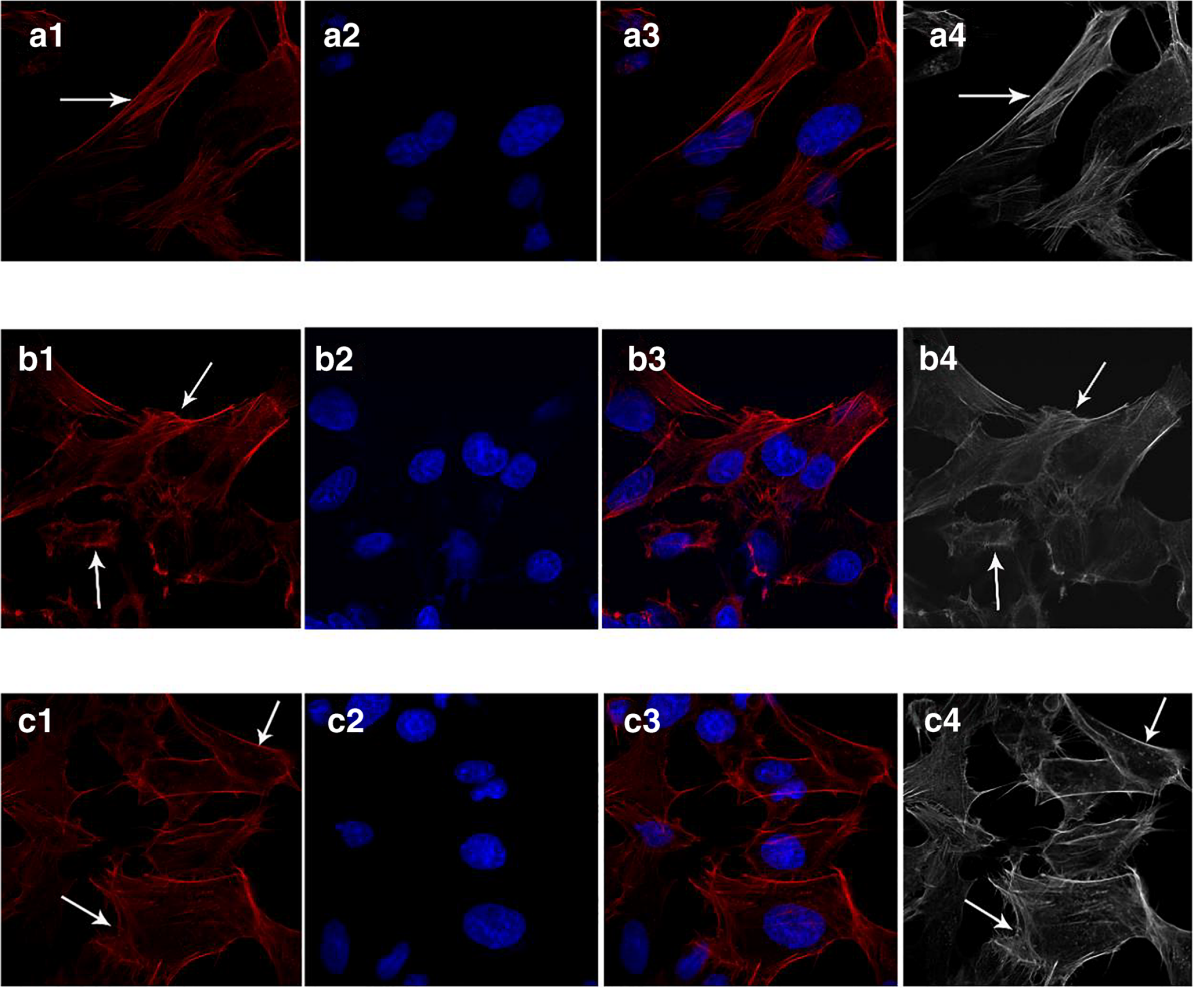
The effects of SA on F-actin expression in HLECs. Control group (a1–4), model group (b1–4), SA treated group (c1–4). Here, a qualitative presentation of the actin cytoskeleton was used confocal microscopy (40 × objective, 1.25 NA)

In the control group, F-actin was distributed regularly and displayed filamentous morphology in the cytoplasm (Fig. 7, a1–4), but in the model group, F-actin was disordered, dispersed and twisted throughout the entire cytoplasm (Fig. 7, b1–4). In comparison to that, the disordered cytoplasm near the cellular periphery was rescued following SA treatment (Fig. 7, c1–4).

## Discussion

LECs play a critical role in the maintenance of lens transparency (Maddala et al. 2020). Lens transparency has been suggested to be associated with mechanical properties of the lens, such as stiffness (Stanga et al. 1999). We have previously shown that SA can improve transparency of lens both in vivo and in vitro (Wei et al. 2012), which we have attributed to an inhibition of aldose reductase activity via impaired transcription and suppression of the polyol pathway (Wu et al. 2016). Nonetheless, the effects of SA on LEC biomechanics remained unclear. Our findings in this study using live cells to demonstrate, for the first time, that SA can improve HLECs function by modulating biomechanics and organelle structure.

Previous studies have suggested that HLECs are highly sensitive to structural damage following high glucose stimulation, but this may be alleviated by inhibition of pathways involved in oxidative stress and the polyol metabolism (Ou et al. 2008; Kubo et al. 1999). Mitochondria are a major source of intracellular reactive oxygen species (Liu et al. 2020). It has been reported that excessive reactive oxygen species may promote cataract formation by disrupting the redox state in lens (Xu et al. 2018), and the accumulation of reactive oxygen species has previously been associated with mitochondrial dysfunction and potential structural damage (Rizwan et al. 2020). Furthermore, endoplasmic reticulum stress, resulting in structural damage, has been observed in LECs in the progression of DC (Periyasamy and Shinohara 2017; Tran et al. 2019). In this study, TEM indicated that SA treatment alleviated structural damage, for example to mitochondria and the endoplasmic reticulum, induced by high glucose stimulation in HLECs.

Cytoskeletal reorganization affects cellular topography, which indicates that changes in mechanical properties of cellular morphology could reflect on structural changes of the cell membrane in HLECs and could help uncover dynamic behaviors of cells (Khandaker et al. 2016; Oprisan et al. 2016; Li et al. 2016). Previously, cytoskeletal changes have been shown to coincide with roughness and stiffness of the cell membrane, which ultimately caused a decrease in cell viscoelasticity and led to changes of cellular biodynamics (Rothdiener et al. 2016; Ge et al. 2016; Gavara 2017). In the present study, AFM showed a greatly increased stiffness in the model group (2.2 increased compared to the control group). Meanwhile, the stiffness after SA treatment was only 1.4 times that of the control group. These real-time observations indicated that SA could reduce the surface roughness and stiffness of the cell membrane of HLECs. Therefore, these results, for the first time, suggest a role of the cellular mechanisms of HLECs in glucose-induced cataract.

In addition to membrane roughness and stiffness, the composition of the phospholipid bilayer also plays a key role in cellular structure and function (Moussa et al. 2017). Raman spectroscopy has previously been applied to detect structural changes of hydrocarbon chains to reveal the characteristics of cellular lipids and phospholipids (Suga et al. 2015). Raman spectroscopy can provide the information on the trans or gauche structure of membrane lipids and the order (S_trans_) and lateral interaction parameter (S_lat_) calculated from it can indicate changes in the membrane liquidity of cells (Anthony 1982). As we demonstrated by Raman spectroscopy and LSCM, the membrane lipid structure and F-actin organization in the cytoskeleton of HLECs was disrupted by high glucose concentrations, but could be rescued by SA treatment. F-actin is one of the integral structure proteins in the membrane, and F-actin changes are closely related to cellular morphology changes (Staszowska et al. 2016; Fong-Ngern et al. 2017). Our results suggested that SA could rescue F-actin disruption and resulted in a more uniform and tight network-like structure of cytoskeleton in high glucose-stimulated HLECs. Combining results from AFM and Raman spectroscopy, these data suggested that a decreased lipid content in the cell membrane reduced cell fluidity, while damage to cytoskeletal proteins increased cellular stiffness and triggered morphological damage (Dawaliby et al. 2016; Needham and Nunn 1990). These processes could ultimately result in a decreased lipid and phospholipid content and increased stiffness of the lens (Zelenka 1984).

## Conclusion

Single-molecule imaging in living cells revealed that SA could rescue the structure of HLECs in the context of high glucose-induced cataract. The increased surface roughness and stiffness of HLECs following SA treatment were due to a decreased cell liquidity and restored F-actin cytoskeletal organization. These results uncovered an effect of SA treatment on the mechanical properties of HLECs, which may ultimately provide a potential new strategy for DC treatment.

## Supplementary Information

ESM 1 (DOCX 12 kb)

ESM 2 (DOC 382 kb)
