# Supplementary material for: Protective effect of syringic acid via restoring cells biomechanics and organelle structure in human lens epithelial cells
Source: J Bioenerg Biomembr. 2021 Mar 11;53(3):275–84. doi: 10.1007/s10863-021-09873-9 (PMC8124055; doi:10.1007/s10863-021-09873-9)
Supplement: Supplementary file 1 — (DOCX 12 kb) [file 10863_2021_9873_MOESM1_ESM.docx]

# Plant authentication and extraction

The stems of D. aurantiacum var. denneanum (kerr) Z.H. Tsi were collected from Lesan County, Sichuan Province, P.R. China, December 2012, and authenticated by Professor Min Li and Tingmo Zhang of the Chengdu University of Chinese Medicine, where a voucher specimen (No. 2012122001) has been deposited in Wan'an Dendrobium Industry and Development Co., Ltd. (Sichuan Province, P.R. China).
